# The Reproduction Number of Swine Viral Respiratory Diseases: A Systematic Review

**DOI:** 10.3390/vetsci11070300

**Published:** 2024-07-02

**Authors:** Dana C. Pittman Ratterree, Sapna Chitlapilly Dass, Martial L. Ndeffo-Mbah

**Affiliations:** 1 Department of Veterinary Integrative Biosciences, School of Veterinary Medicine and Biomedical Sciences, Texas A&M University, College Station, TX 77843, USA; dana.pittman@tamu.edu; 2 Department of Animal Science, College of Agriculture and Life Sciences, Texas A&M University, College Station, TX 77843, USA

**Keywords:** reproductive number, swine diseases, viral respiratory diseases, within-herd transmission, between-herd transmission

## Abstract

**Simple Summary:**

Diseases in the swine industry can cause major economic and health issues. This review examines R_0_ estimates, which measure how efficiently respiratory diseases spread among pigs and compares transmission risks within and between farms. Seven diseases were studied: Aujeszky’s disease, PPRSV, Circovirus, Influenza A, Encephalomyocarditis Virus, Classical Swine Fever, and African Swine Fever. Data from research articles showed varying R_0_ values, with higher transmission rates within herds for some diseases. Effective disease control requires prompt detection, response to outbreaks, and continuous monitoring to reduce its impact on the swine industry.

**Abstract:**

Diseases in the swine industry can cause significant economic and health impacts. This review examines R_0_ estimates for respiratory diseases in pigs, assessing variations and comparing transmission risks within and between farms. A literature search of three databases aggregated peer-reviewed research articles on swine viral respiratory diseases’ R_0_ values. The study focused on seven diseases: Aujeszky’s disease (AD), Porcine Reproductive and Respiratory Syndrome Virus (PRRSV), Circovirus, Influenza A (IA), Encephalomyocarditis Virus (EV), Classical Swine Fever (CSF), and African Swine Fever (ASF). R_0_ values were estimated for transmission within and between herds/farms using various methods, from complex mathematical models to simple calculations. Data primarily came from disease surveillance and laboratory experiments. The median R_0_ for within-herd and between-herd transmission was 10 and 3.31 for AD, 2.78 and 1.14 for PRRSV, 5.9 and 0.89 for Circovirus, 1.75 and 1.6 for CSF, and 3.94 and 3.15 for ASF. For IA and EV, only within-herd R_0_ values were estimated at 8.65 and 1.3, respectively. Diseases with high R_0_ values highlight the need for prompt detection and response to outbreaks. Continuous monitoring and evaluation of pathogen transmissibility are crucial for enhancing disease surveillance and reducing the impact of livestock diseases.

## 1. Introduction

Pork is a leading source of protein consumption worldwide, making up a third of all meat production globally [1]. Therefore, diseases in the swine industry are likely to have significant economic and indirect health impacts in both pork-producing and pork-consuming communities. A disease is considered economically important if it leads to death, wasting, decreased efficiency of gain, reduced rate of gain, immune suppression/increased susceptibility to other disease conditions, or infertility in livestock species used as food [2,3]. Respiratory diseases are one of the most important causes of economic losses in pork production [2,3,4].

In this study, we review seven viral diseases with various respiratory clinical presentations and transmission routes. Respiratory symptoms include but are not limited to coughing, sneezing, shortness of breath (dyspnea), and inability to grow up to production standards [1,5,6]. Aujeszky’s disease, caused by suid herpesvirus 1, is a respiratory syndromic disease that can be spread by various transmission routes, including direct contact, airborne, and fomite transmission [7]. While the disease has been mostly eradicated in many countries in Europe and North America, it remains endemic in areas like south-eastern Europe and Latin America [8]. Circoviruses, such as Porcine Circovirus 2 and 3 (PCV2 and PCV3), are emerging pathogens in the pork industry that impact multiple organ systems, including the respiratory system [9,10]. Studies indicate that PCV2 has been detected in air samples, and evidence indicates that there is short-distance transmission [11,12]. Encephalomyocarditis in pigs is best categorized as a respiratory syndrome, as transmission occurs primarily through contaminated feed and water [13,14]. African Swine Fever (ASF) was believed to be primarily transmitted through secretion and only has a clinical respiratory manifestation, but recent research has evidence of aerosol transmission through short distances [15,16]. Like ASF, Classical Swine Fever has evidence of short-distance aerosol transmission especially high-virulence strains [17,18,19]. Both Influenza A Virus (IAV) and Porcine Reproductive and Respiratory Syndrome Virus (PRRSV) spread through aerosol transmission, and the disease impacts gain, often leading to death in piglets [20,21,22,23,24]. While acute and dramatic clinical signs may resolve relatively quickly, this does not mean that their subclinical effects and costs are self-limiting or resolve quickly. Because pork makes up a significant portion of global meat production, research on swine diseases has been primarily driven by the needs of the food industry [1].

The basic reproductive number (R_0_) is the expected number of secondary infections generated by an infectious individual in an otherwise susceptible population. When R_0_ is less than one, the disease outbreak dies off as each infectious individual produces less than one new infection on average. Conversely, when R_0_ is above the threshold value, each infection generates multiple new infections, allowing the disease to spread through a population. This epidemiological metric has been widely used to inform the risk and potential magnitude of a disease outbreak, the critical herd size required for pathogen persistence, and the efficacy or magnitude of control efforts [1,25]. This review summarizes R_0_ estimates of respiratory diseases in pigs, as reported in the literature, evaluates variations in reported R_0_ values, and compares estimates of disease transmission risk within and between pig farms.

## 2. Materials and Methods

A literature search was conducted in the PubMed, Web of Science, and EMBASE (OVID) databases to aggregate peer-reviewed original research articles on R_0_ of pig viral respiratory diseases. The PRISMA guidelines were utilized to identify, screen, and determine the eligibility of the articles. A list of diseases was identified by searching the Merck Veterinary Manual and CABI Compendium by combining the terms “respiratory disease” and “pigs” [6,26]. Search terms were aggregated into disease and reproductive number categories. The terms “swine”, “pig”, and “porcine” were used to narrow the study populations. Specific viral diseases and general terminology for respiratory diseases were combined to make a disease query. The full list of search queries is provided in Table 1. All citations were imported into EndNote 21 (EndNote Version 21.2, Clarivate, Philadelphia, PA, USA).

The search returned a combination of modeling exercises, laboratory experiments, and observed outbreaks. The exclusion criteria were as follows: bacterial diseases, studies that only reported the reproductive number for intervention strategies (i.e., vaccination), research not classified as original research, and articles not published in English. To be included, the paper had research that applied to domestic pig production. From the remaining studies, information on herd classification, modeling approach, and method of calculating R_0_ was extracted. The herd classifications were within- and between-herd to specify whether a paper-modeled disease spread within a single farm population or across multiple farms. A brief statement about the type of model used in each paper was created from their method section.

## 3. Results

**Figure 1:**
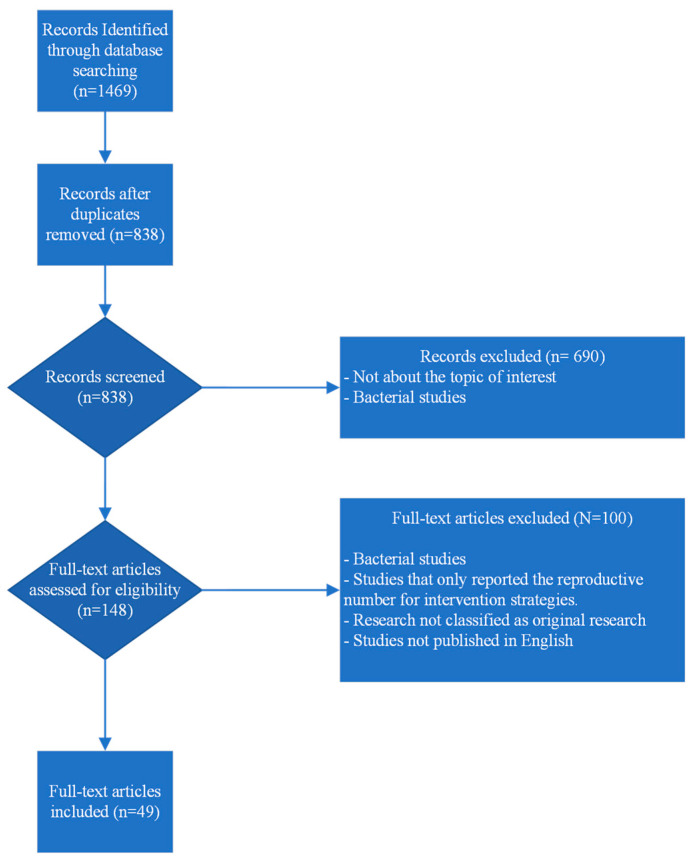
PRISMA chart of Pig Viral Respiratory diseases.

**Figure 2:**
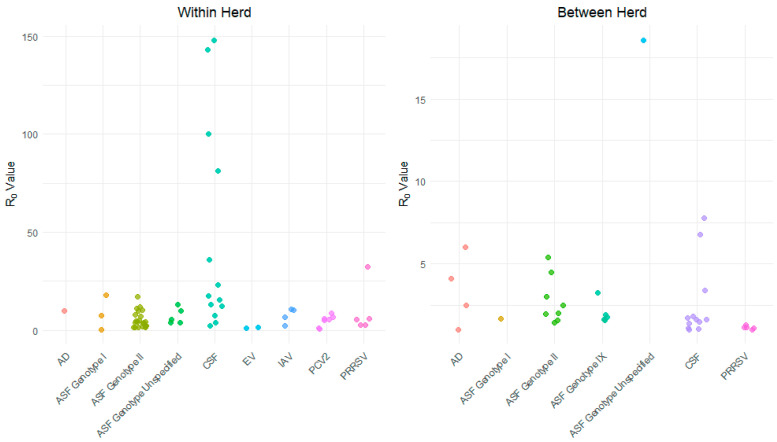
Distribution of R_0_ values by disease for within- and between-herd transmission.

The total number of papers identified was 1469, of which 631 were duplicates, leaving 838 studies. After screening the titles and abstracts, 148 papers were selected for full-text screening. Among these, 49 papers covering seven diseases were deemed eligible for inclusion in the review (Figure 1). R_0_ values were estimated for disease transmission within herds/farms and between herds/farms (Figure 2). Various methods were used in these papers to calculate the R_0_ values, ranging from complex mathematical approaches like the Next-Generation method to simple direct calculations of the number of secondary infections from simulation models. The most commonly used method to calculate R_0_ for a specific disease model was the product of the disease transmission rate and duration of infection (Table 2). These parameters were estimated through model fitting to reported case data or directly inferred from laboratory experiments.

**Figure 3:**
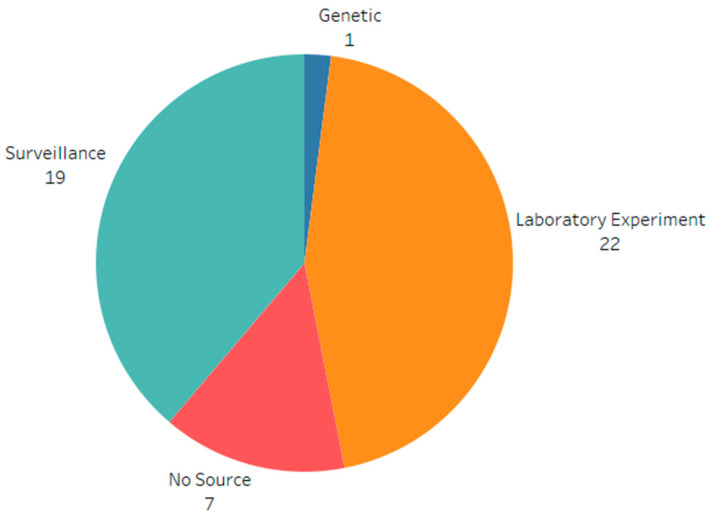
Distribution of data types extracted for all swine respiratory diseases.

The data used in these studies for calibrating the models primarily consist of surveillance data and laboratory experiments. Surveillance data, as defined in this review, refers to data from a secondary source, such as a government institution in a specific geographic region. In contrast, laboratory experiments were conducted to investigate disease transmission in a controlled environment. Nine studies did not use empirical data to parameterize their models or did not explicitly specify the sources of the data or values. A phylogenetic study utilized genetic data from previous outbreaks to calculate the reproductive number. The counts and distribution of the data types are shown in Figure 3.

### 3.1. Aujeszky’s Disease

Three publications evaluated the R_0_ value of Aujeszky’s disease (Table 2). Out of these three publications, two developed a generic pig population to model Aujeszky’s disease transmission, and one developed a model for Aujeszky’s disease outbreaks in the Netherlands [27,28,29]. All three studies used stochastic compartmental models of disease transmission [27,28,29]. The two generic population models estimated the R_0_ value to be 6 and 4.12, using the Next-Generation Matrix method and direct calculation from model simulations, respectively [27,29]. In the Netherlands study, the Next-Generation Matrix method was used to compute the between-herd R_0_ value, and a survival function method was used for the within-herd R_0_ value [28]. The between-herd R_0_ value was estimated to range between 1 and 2.5, and the within-herd R_0_ value was equal to 10 [28]. The within-herd R_0_ value of 10 was three times as high as the median between-herd R_0_ value of 3.31 (Table 2).

### 3.2. Porcine Circovirus 3

Only one study met the inclusion criteria for Porcine Circovirus 3. The study by Li et al. identified two major genotypes for PCV3 and estimated the R_0_ value to be 3.08 (95% credible interval (CrI): 0.82–7.96) and 1.82 (95% CrI: 0.63–4.82) for PCV3a and PCV3b, respectively [10]. The phylogenetic analysis used for R_0_ value calculations was based on a birth-death skyline model, and R_0_ was calculated as the product of the transmission rate and duration of infection [10].

### 3.3. Porcine Circovirus 2

Three studies from the same research team estimated R_0_ for Porcine Circovirus 2 (PCV2). Andraud et al. calculated the R_0_ value within and between pens for both types of within-herd transmission [30]. They calculated R_0_ under two different assumptions about infectiousness: (1) Infectiousness ends at seroconversion, and (2) Infectiousness ends at seroconversion, and viral load decline occurs. Under assumption 1, the between-pen R_0_ was estimated to be 0.58 (95% Confidence Interval (CI): 0.23–1.47), and under assumption 2, between-pen R_0_ increased to 1.2 (95% CI: 0.5, 2.9). The within-pen R_0_ was estimated to be 5.5 (95% CI: 3.3–9.0) under assumption 1 and 8.9 (95% CI: 5.1–15.4) under assumption 2 [30]. The study developed a stochastic compartmental model for PCV2 transmission parameterized using animal experiment data. R_0_ was calculated as the product of the transmission rate and duration of infection [30]. Another study by Andraud et al. used a deterministic model, parameterized using animal experiment data, to estimate the within-herd transmission of PCV2 [31]. The R_0_ value was 5.9 (95% CI: 1.8–10.1), estimated using a survival function method [31]. The last study used two experimental datasets to estimate the within-herd R_0_ value using a final-size algorithm [32]. These were time-series individual-level virological data collected from laboratory experiments to investigate PCV2 transmission in controlled environments. The first study was conducted by Rose et al., and the second by Andraud et al. [30,32]. Using the data produced by their experiment, Rose et al. calculated R_0_ as 6.94 (95% CI: 0.42–15.01) [32]. By merging their data with Andruad et al., a previous experiment by the same research group, R_0_ was estimated as 5.1 (95% CI: 2.5–8.2) [30,32].

### 3.4. Influenza A

Three publications estimated the within-herd R_0_ value of swine Influenza A (Table 2). Rose et al. used case data from outbreaks on swine farms in Brittany, France, and deterministic compartmental models to investigate the dynamics of within-farm swine Influenza outbreaks [5]. They used Poisson regression to fit their models to case incidence data and an exponential growth method to calculate the R_0_ value. The within-herd R_0_ value was estimated to range from 2.5 to 6.9 [5]. The remaining two papers used similar study designs and generated comparable results. These similarities include using data from animal experimental trial studies, stochastic compartmental models for disease transmission, and calculating R_0_ as the product of the transmission rate times the duration of infection (Table 2). Their R_0_ values of 10.4 and 10.66, respectively [33,34], were significantly lower than those reported by Rose et al. [5].

### 3.5. Porcine Reproductive and Respiratory Syndrome Virus

For Porcine Reproductive and Respiratory Syndrome Virus (PRRSV), six publications estimated the within-herd R_0_ to range from 2.6 to 5.42 (Table 2). Four studies used a generic pig population to model PRRSV outbreaks, and two modeled PRRSV outbreaks in the United States (Table 2). Of the generic population studies, three used laboratory experiments to inform parameter values of compartmental models and calculated R_0_ as the product of the transmission rate times the duration of the infection. The fourth study used the survival function method to calculate within-herd R_0_.

Two studies estimated the between-herd R_0_ in the United States using data from the Morrison Swine Health Monitoring Project (MSHMP) and transmission trees to model disease spread [35,36]. Pamornchianavakul et al. used phylogenetic analysis, while Arruda et al. utilized the Wallinga and Teunis method to calculate R_0_ values [35,36]. Both estimated between-herd R_0_ values of around 1 despite using different calculation methods (Table 2 [35,36]).

### 3.6. Encephalomyocarditis

Two publications estimated the within-herd R_0_ for encephalomyocarditis. Both studies used a stochastic compartmental model to investigate within-farm disease transmission and the White and Pagano method to compute R_0_ (Table 2). The first study by Kluivers et al. used serological data from infected pens on a pig farm during an autumn 2001 outbreak in Belgium to inform their model [13,14], and the second study by Maurice et al. used data from animal laboratory experiments. Kluivers et al. estimated R_0_ as 1.36 (95% CI: 0.93, 2.23), and Maurice et al. estimated it as 1.24 (95% CI: 0.39, 4.35) [13,14].

### 3.7. Classic Swine Fever

Twelve publications evaluated the R_0_ value of Classical Swine Fever (CSF) (Table 2). Eight of the twelve papers used generic populations to model CSF transmission; two papers used case data from the Netherlands, one used data from India, and one used data from Bangladesh (Table 2). Six studies calculated R_0_ as the product of transmission rate times duration of infection; two used an attack rate method, two used an exponential growth method, and two used a martingale estimator method (Table 2). 

A machine learning model was developed by Suresh et al. to create an early warning system for potential outbreaks related to climate change [42]. The model was trained on geotagged incidence data for Guwahati City, Assam, from 2005 to 2021 [42]. Risk factors, such as humidity and vegetation, were added to the model to ascertain the climate-disease relationship on a spatial grid. R_0_ was calculated using the attack rate for each grid, producing values ranging from 1.04 to 2.07 [42]. Chowdhuary et al. used an exponential growth function to model surveillance data for a 2015 outbreak in the Kurigram district of Bangladesh [41]. They used two methods for calculating R_0_: the attack rate and the exponential growth rate (Table 2). The attack rate method estimated R_0_ as 1.6 (95% CI: 1.5–1.7) and exponential growth estimated as 1.5 (95% CI: 1.3–1.7) (Table 2). 

Stegeman et al. published two papers using data from the 1997 to 1998 outbreak of CSF in the Netherlands to estimate the within- and between-herd R_0_ (Table 2). The between-herd R_0_ was estimated to be 6.8 [43]. Data were obtained from 21,500 herds in the Netherlands, of which 429 experienced an outbreak. In the second paper, a simple deterministic compartmental model was fitted to the data, and the within-herd R_0_ was calculated as the product of the transmission rate times the duration of infection. To calculate the within-herd R_0_, Stegeman et al. used data from 82 of the 429 infected herds because these outbreaks could be identified from a single known contact [44]. The within-herd R_0_ was estimated at 2.9, using the exponential growth method applied to a stochastic compartmental model [44]. 

Durand et al. investigated the potential use of clinical data (quantitative and qualitative), considered as infectivity markers, to reliably estimate CSF R_0_ value. The clinical-qualitative dataset reported the number of infected animals with clinical scores greater than 0, and the clinical-quantitative dataset reported the sum of clinical scores of infected animals [48]. They used viremia-based data (virus in the bloodstream) as the standard dataset for R_0_ calculation [48]. R_0_ values computed from the clinical datasets were higher than the viremia-based ones, but the confidence intervals all overlapped, indicating that these values were not significantly different (Table 2). The within-herd values varied from 4.0 (95% CI: 1.9–8.4) to 12.2 (95% CI: 5.5–27.3), and the between-herd values varied from 1.1 (95% CI: 0.4–2.9) to 1.8 (95% CI: 0.7–4.8) (Table 2) [48]. 

Laevans et al. performed two animal experimental studies using pigs of different ages to evaluate the within-pen risk of CSF transmission [25,26]. They used experimental data with different final outbreak size methods for R_0_ calculation to estimate within-herd R_0_ values [18,19]. The weaner pigs had an estimated R_0_ of 81.3, whereas the slaughter pigs had a significantly smaller estimate of 13.7 [18,19]. Klinkenberg et al. used data collected in the Laevans et al. experiments to parameterize a stochastic compartmental model of CSF transmission [18,19,45]. Calculating R_0_ as the product of the transmission rate times duration of infection, they estimated the within-herd R_0_ to be 100 for weaner pigs and 15.5 for the slaughter pigs; these estimates were much higher than those of Laevans et al. (Table 2). These results clearly illustrate the high sensitivity of R_0_ to the calculation method.

Weesendrop et al. reported the highest within-herd R_0_ values of 143 for a high transmissibility strain and 23 for a low transmissibility strain [49]. Bouma et al. reported the lowest within-herd R_0_ value, 2.3 (95% CI: 0–5.0) [46]. This disease had the largest range of R_0_ estimates across all diseases, likely due to the diversity in the R_0_ calculation methods and the type of data used. Studies using laboratory experimental data were more likely to generate higher R_0_ values than those using surveillance data (Table 2).

### 3.8. African Swine Fever

Eighteen publications estimated R_0_ for African Swine Fever (ASF), the most common disease identified in this search (Table 2). Among the articles extracted, there was an even split between studies using generic populations to model ASF transmission and those using laboratory experiment data and location-specific surveillance data. Nine studies utilized surveillance data to calibrate their models, four studies used data from Vietnam, two studies used data from China, two studies used data from Russia, one study used data from Ukraine, and one study used data from Uganda (Table 2). Fourteen studies estimated within-herd R_0_, and five studies estimated between-herd R_0_. Using genotyping information from surveillance data, R_0_ results were stratified by genotype.

#### 3.8.1. Genotype IX

Only Barongo et al. investigated genotype IX, a strain mostly found in eastern Africa [51]. This Uganda study focused on small free-range pig production in the Gulu district, whose economy is rooted in small-scale agriculture [51]. The data used in this study were part of a larger effort to understand the transmission of ASF in Gulu District [51]. The period of interest was from April 2010 to November 2011 [51]. Herds were defined as all pigs in a pig-keeping household [51]. Barongo et al. used three separate models to depict transmission: a spatial simulation, an epidemic doubling time method, and a simple SI (Susceptible-Infected) deterministic model. The spatial simulation, referred to as the nearest infectious neighbor by Barongo et al., had the highest estimate of R_0_ as 3.24 (95% CI: 3.21–3.27) [51]. The doubling time method produced an R_0_ of 1.63. For the SI model, R_0_ was computed as the product of the transmission rate and duration of infection, and the model was fitted to the data in three ways: linear regression, curve fitting, and bootstrapping, leading to slightly different R_0_ values. The within-herd R_0_ was estimated as 1.58 for curve fitting, 1.77 for bootstrapping, and 1.9 for linear regression [51].

#### 3.8.2. Genotype I

R_0_ estimates for genotype I were calculated in two laboratory experiments and in one study using surveillance data from Ukraine. Eble et al. used data from a previous laboratory experiment for the Netherlands/86 strain to calculate the R_0_ for carrier animals, finding that transmission does occur but at low levels with R_0_ less than 1 (R_0_ = 0.3) [52]. De Carvalho Ferreira et al. generated R_0_ estimates of 18.0 (95% CI: 6.90–46.9) for the Malta/78 isolate and 4.92 (95% CI: 1.45–16.6) for Netherlands/86 [53]. The Ukraine study investigated the transmission of ASF in the Odesa region of Ukraine [54]. It used historical data from the 1977 outbreak collected by the USSR Ministry of Agriculture [54]. The study estimated the between and within-herd R_0_ to be 1.65 (95% CI: 1.42–1.88) and 7.46 (95% CI: 5.68–9.21), respectively [54].

#### 3.8.3. Genotype II

The most recent R_0_ estimates come from outbreaks in Asia. Of the 11 studies of genotype II ASF, three used data from outbreaks in China and four studies used data from Vietnam. All the studies out of Asia pertain to the introduction of the disease to the country, which occurred from late 2018 to early 2019 [55,57,58,59,61,62,63].

All studies on ASF in China used deterministic compartmental models and the Next-Generation Matrix method to estimate R_0_. Haung et al. used incidence data from the Ministry of Rural Agriculture of China and estimated R_0_ to be 0.6 [55]. Song et al. used data from the August 2018 ASF outbreak at an Aiyuan farm of Jiangsu Jiahua Breeding Pig Company in Siyang County, China [58]. The R_0_ value was estimated to be 4.18, which was smaller than that reported by Li et al. (Table 2) [58,59]. Li et al. used data from outbreaks in Xuanzhou at Guquan, Jinba, and Liacheng facilities during September 2018 and estimated R_0_ as 4.82 (95% CI: 3.84, 6.11) in Guquan, 7.94 (95% CI: 7.26, 8.72) in Jinba, and 11.90 (95% CI: 10.71, 12.91) in Liancheng [59].

All four studies pertaining to Vietnam used data from the 2019 outbreak of ASF in the northern region, the introduction of the disease into the country. Three of the four used compartmental models, and one used a serial interval model assuming a Poisson distribution to model daily incidence. Mai et al. was the only study that calculated the between-herd R_0_ [57]. The between-herd R_0_ was calculated for 15, 19, and 30-day infectious periods, yielding a range of values from 1.41 to 10.8 [57]. Table 2 shows the between-herd R_0_ values at different locations for the 15-day infectious period. Mai et al. used three methods to estimate the within-herd R_0_ using data from seven farms in Hung Yen and three farms in Ninh Binh and Ha Nam in Vietnam [62]. They estimated R_0_ as 1.49 using the exponential growth method, 1.58 using the White and Pagano method, and 1.46 using the attack rate method [62]. These data were also used to parameterize a SIR (Susceptible-Infected-Recovered) model under two farm sizes and lengths of the infectious period. They estimated R_0_ for a 100–299 pig farm to be 1.66 (95% CI: 0.88–2.84) and 1.4 (95% CI: 1.01–1.90) for a 300–999 pig farm [62]. Another study used only two mid-sized farms from Hung Yen using a statistics-based model [57]. These facilities were chosen for commercial design, strict biosecurity measures, and the way workers were assigned to only work in one barn with no visitors allowed on the premises [63]. Le et al. collected data from 15 March through 15 November 2019, on a small family-owned farm in Hanoi, Vietnam, that only had seventeen pigs [61]. They estimated the within-herd R_0_ to be 10.4 (95% CrI: 1.1–30.4) [61]. The remaining two studies estimated R_0_ to range from 1.55–1.78 [57,62].

The two studies from Russia used compartmental models and data from the Caucasian region and calculated R_0_ as the product of the transmission rate and the duration of the infection. Guinat et al. used data from the Federal Research Center for Virology and Microbiology and selected data from nine industrial herds between 2010 and 2014 [60]. A stochastic modeling approach was implemented to estimate the within-herd transmission of the infection [60]. The smallest within-herd R_0_ median value was estimated as 4.4 (95% CrI: 2.09–13.4) and the largest as 17.3 (95% 3.5–45.5) [60]. Gulenkin et al. obtained data from the World Animal Health Information System Office International Epizootic (WAHID OIE) database for the period between 2007 and 2010 [56]. They used a deterministic model for both between and within-herd disease transmission. Using a curve fitting method to estimate the model parameters, the within-herd R_0_ was estimated to range from 8 to 11. Alternatively, using a general linear model for model fitting R_0_, the value was estimated as 9.8 (95% CI: 3.9–15.6) [56]. Estimates for the between-herd R_0_ were limited to data from the Republic of North Ossetia in 2008, and its value ranged from 2 to 3 [56].

Two studies related to ASF genotype II used laboratory experiments to model within-herd ASF transmission. Guinet et al. utilized compartmental models, and the product of the transmission rate times duration of infection was the most used approach to calculate R_0_. Guinet et al. conducted an experiment on the Georgia 2007/1 strain and used a stochastic model considering different latent periods [64]. They calculated R_0_ for within and between pens for 3-, 4-, and 5-day latent periods. Oh et al. inoculated pigs using ASF from a 2020 outbreak in Thanh Hóa province, Vietnam, using survival analysis to model transmission between pigs [65]. R_0_ was calculated using the exponential growth method and White and Pagano method, producing the values 2.91 (95% CI: 1.51–7.06) and 4.015 (95% CI; 1.13–9.8), respectively.

#### 3.8.4. Genotype Not Specified

Five papers produced mathematical models developed to analyze the dynamics of ASF outbreaks but did not specify the genotype used in parameterization. Parameters for these models come from the literature; therefore, these models are proof of concept rather than applied to specific outbreaks [68,69,70]. Four of the five studies estimated within-herd R_0_ ranging from 3.77 to 13.02. The fifth study developed a model to analyze the spread of ASF caused by a contaminated human vector between farms, calculating R_0_ as 18.57 [66].

## 4. Discussion

### 4.1. R_0_ Methodologies

The methods used for calculating R_0_ varied greatly between studies. This review found 16 different ways R_0_ was calculated, with each disease having at least two different calculation methods. Calculation methods ranged from simple, like the product of the transmission rate and the duration of infection, to more complex methods, like the Next-Generation Matrix. The measure of R_0_ itself is situation-specific. Studies using data from animal experimental trials tended to use the product of the transmission rate times the duration of the infection, while studies using surveillance data employed the largest range of calculation methods, with most studies using more than one method to estimate R_0_. For example, Chowdhuary et al. used both attack rate and exponential growth methods to estimate the R_0_ for CSF and found no significant differences between the estimates [41]. On the other hand, Barongo et al. used four methods for calculating R_0_, with one of the estimates above 3 and the rest between 1.5 and 2 [51]. Modeling studies using complex compartmental models for disease transmission have tended to use the Next-Generation Matrix method for R_0_ calculation. Differences in R_0_ estimates for the same pathogen may vary with R_0_ calculation methods, pathogen variants [37,71], the data source used for model calibration (surveillance vs. laboratory data), and data location. 

### 4.2. Comparison of R_0_ Estimates from Laboratory and Surveillance Data

Laboratory experiments often yield higher R_0_ estimates compared to surveillance data for several reasons. It must be noted that laboratory experiments are limited in scope, as they can primarily be used to calculate within-herd transmission for a relatively small number of animals. In contrast, surveillance data are broad and can encompass not only the incidence on one farm but an entire geographic area, covering hundreds or thousands of animals. Due to the magnitude of farms, surveillance data may underreport cases as animals are not continuously tested, whereas the controlled environment of laboratory experiments allows for animals to be continuously tested. Furthermore, the environment of laboratory experiments lends itself to an increased transmission risk. Experiments are often conducted in relatively small pens or rooms, potentially enhancing disease transmission compared to actual farms that generally have larger pens or rooms. Laevans et al. attributed differences in pen size for weaner and slaughter pigs as the primary factor leading to a significant magnitude difference between the two estimates, 81.3 for weaner pigs and 13.7 for slaughter pigs [18,19]. Additionally, in experimental trials, the control group is not subject to any disease mitigation measures, whereas farms, especially industrial ones, continuously employ some level of biosecurity or mitigation measures. Some experiments in this review utilized high-virulence strains, which may not reflect the strains currently circulating on farms, potentially leading to an overestimation of R_0_ and misrepresentation of actual transmission risks [49,50,53].

### 4.3. Comparison of within and Between-Herd R_0_

Understanding the transmission dynamics of viruses involves examining two main aspects: within- and between-herd R_0_. Between-herd R_0_ tends to show lower values compared to within-herd R_0_, partly due to how it is measured. The between-herd R_0_ estimates the virus’s ability to spread from one farm to another, essentially tracking its movement between different populations in distinct areas. The unit of measurement here is a farm, regardless of its pig population size. Conversely, within-herd R_0_ focuses on the virus’s transmission from pig to pig within a single farm. This often yields higher estimates, possibly because there are more opportunities for interactions among animals within the same farm compared to interactions between animals from different farms, such as through animal movement. Consequently, diseases like viral respiratory illnesses are more likely to spread rapidly within a single farm than between different farms. When calculating within-herd R_0_, we typically rely on data about the incidence of infection within a specific farm, while between-herd R_0_ is based on information about the number of farms infected over time.

### 4.4. Disease Eradication and R_0_

The basic reproduction number is a key epidemiological metric that can be used to measure the transmission risk of infectious disease, predict the expected size of an outbreak, and estimate the control effort needed to prevent or interrupt disease transmission, among others [72]. However, the R_0_ value is not necessarily a direct indication of the potential eradication of an animal infectious disease. Disease eradication depends on several factors, such as disease characteristics (e.g., host reservoirs, clinical symptoms, infectious period, and immunity duration), economic factors, political will, and control tools (e.g., vaccine, depopulation, culling, movement restrictions) [73,74]. For example, an animal disease with a low R_0_ value, several host species, and/or wildlife reservoirs, such as Circovirus infection, may be harder to eradicate than a disease with a higher R_0_ value but limited to a single host species with no persistence in wildlife, such as AD.

### 4.5. Limitations of R_0_ Calculation for Complex Diseases

Diseases such as PRRSV have a complex infectious period, during which infectious hosts experience both acute and subclinical/chronic infectious phases with different levels of infectivity. Although this characteristic is central to PRRSV disease progression and transmission risk, most PRRSV R_0_ models have not explicitly considered the differential role of these infectious phases in disease transmission, and none has estimated their contribution to R_0_ value [7,35,36,37,38,39,40,75,76]. Such information would be pivotal for accurately evaluating the timing and effectiveness of disease control strategies. Further studies should investigate the differential contribution of acute and subclinical/chronic infectious phases to R_0_ and develop a general framework to deal with these situations in computing R_0_ and other disease transmission risk metrics.

## 5. Conclusions

Despite the limitations of R_0_, it is a useful tool to begin quantifying the risk of disease outbreaks and the magnitude of an outbreak and investigate intervention strategies’ effectiveness for disease control or prevention. For example, understanding the R_0_ for within-herd transmission is crucial for determining the vaccination efficacy required for preventing or eradicating disease on a farm. Between-herd R_0_ is paramount for understanding the vulnerability of farm networks to disease outbreaks and the vaccination coverage required to prevent large-scale disease outbreaks. Between-herd transmission is mostly driven by interactions between farms through commerce, supply chains, and the movement of infected livestock and related products. To improve the estimate of between-herd R_0_, future studies should explicitly incorporate data on interactions between farms. However, such interaction and mobility datasets are not readily available for most countries. 

Swine diseases with a larger R_0_ emphasize the urgency of promptly detecting and responding to outbreaks. Addressing outbreaks can be challenging, regardless of resource availability. Through timely case surveillance, forecasting tools, and rapid dissemination of information to farmers, veterinarians, and government officials, animals and the food supply can be better protected against infectious disease outbreaks. Preventing swine respiratory diseases from spreading and entering the global supply chain is imperative, as they pose an imminent threat to food supply. Continuous monitoring and evaluation of pathogen transmissibility are paramount to enhance disease surveillance efficiency and decrease the impact of livestock diseases.

## Figures and Tables

**Table 1:** Terms by category used for generating results.

Category	Terms
Mathematical	“Reproduction number” OR “reproductive number” OR “reproductive rate” OR “reproductive ratio” OR “reproduction ratio” OR “R0” OR “force of infection” OR “secondary infection” OR “secondary case” OR “attack rate” OR “attack ratio” OR “population dynamics” OR “infection dynamics” OR “transmission rate” or “R0 Structural Parameters”
Animal	livestock OR farm * OR pig OR porcine OR swine
Disease	“Respiratory virus” OR “Respiratory disease” OR paramyxovirus OR herpesvirus OR alphaherpesvirus OR “adenovirus” OR “viral pneumonia” OR “porcine respiratory disease” OR “porcine infectious disease” OR “porcine coronavirus” OR “porcine respirovirus 1” OR “swine orthopneuovirus” OR “swine influenza” OR “Porcine reproductive and respiratory virus” OR PRRSV OR “syndrome PRRSV Virus” OR “swine influenza” OR SIV OR “African Swine Fever” OR ASF OR ASFV OR “suid herpesvirus 1” OR SHV1 OR “Porcine Epidemic Abortion and Respiratory Syndrome” OR “classical swine fever” OR “CSF” OR “H1N1”OR “H3N2” OR “H1N2”OR “PRCV” OR “PCV2” OR “porcine cytomegalovirus” OR “PCMV”OR “Aujeszky’s disease virus” OR “ADV”OR “Encephalmyocarditis Virus” OR “hemagglutinating encephalomyelitis virus”

The asterisk is a wildcard search operator to include different ending of the word like “farming” and “farms”.

**Table 2:** Extraction results stratified by disease.

Study	R_0_	Herd Type	Location	Method	Model	Data Source
Aujeszky’s Disease/Pseudorabies						
(Houben et al., 1993) [27]	6	Between	Generic	Next-Generation Method	Stochastic compartmental model	No empirical data used
(Buijtels et al., 1997) [28]	4.12	Between	Generic	Estimated directly from model simulation	Stochastic compartmental model	No empirical data used
(Van Nes et al., 1998) [29]	1–2.5	Between	The Netherlands	Next-Generation Method	Stochastic compartmental model	Empirical case data from one of the largest breeding companies in the Netherlands and the Central Bureau of Statistics.
(Van Nes et al., 1998) [29]	10	Within	The Netherlands	Survival Function Method	Stochastic compartmental model	Empirical case data from one of the largest breeding companies in the Netherlands and the Central Bureau of Statistics.
Porcine Circovirus 3						
(Li et al., 2018) [10]	3.08 (CrI: 0.82–7.96)	Not specified	Global	Product of the transmission rate and duration of infection	Birth-death skyline model	Gene sequences data
(Li et al., 2018) [10]	1.82 (CrI: 0.63–4.86)	Not specified	Global	Product of transmission rate and duration of infection	Birth-death skyline model	Gene sequences data
Porcine Circovirus 2						
(Andraud et al., 2008) [30]	Between pen 1: 0.58 (CI: 0.23–1.47) Between pen 2: 1.2 (CI: 0.5–2.9) Within pen 1 5.5 (CI: 3.3–9.0) Within pen 2 8.9 (CI:5.1–15.4)	Within	Generic	Product of transmission rate and duration of infection	Stochastic compartmental model	Animal experiments conducted by the authors (France)
(Andraud et al., 2009) [31]	5.9 (CI: 1.8–10.1)	Within	Generic	Survival Function Method	Deterministic compartmental model	Animal experiments conducted by the authors (France)
(Rose et al., 2016) [32]	5.1 (CrI: 2.5–8.2) 6.94 (CrI: 0.42–15.05)	Within	Generic	Final-Size Algorithm	Exponential decay function to model the individual probability of escaping infection	Animal experiments conducted by the authors (France)
Influenza A						
(Allerson et al., 2013) [33]	10.4 (CI: 6.6–15.8)	Within	Generic	Product of transmission rate and duration of infection	Stochastic compartmental model	Animal experiments conducted by the authors (United States)
(Rose et al., 2013) [5]	2.5 (CI: 1.9–2.9) to 6.9 (CI: 4.1–10.5)	Within	France	Exponential growth Method	Deterministic compartmental model	Cases data of outbreaks in swine farms located in Brittany, France
(Romagosa et al., 2011) [34]	10.66 (CI: 6.57–16.46)	Within	Generic	Product of transmission rate and duration of infection	Stochastic compartmental model	Animal experiments conducted by the authors (United States)
PRRSV						
(Pamornchainavakul et al., 2023) [35]	1 (IQR: 1–2). Highest value of 5	Between	United States	Phylogenetic analysis	Transmission tree model	Morrison Swine Health Monitoring Project (MSHMP) database, which was established to track progress on PRRS control in the U.S. and aimed to be the national hub for voluntary data sharing between swine veterinarians from different production systems
(Arruda et al., 2017) [36]	Southeast: 1.14, South: 1.14, Upper Midwest East: 1.30, Upper Midwest West: 1.10.	Between	United States	Wallinga & Teunis Method	Transmission tree model	MSHMP database, which was established to track progress on PRRS control in the U.S. and aimed to be the national hub for voluntary data sharing between swine veterinarians from different production systems. Four areas across the US were chosen, including farms located in the states of North Carolina [Southeast (SE)], Oklahoma [South (S)], Minnesota/Iowa [Upper Midwest East (UME)], and Nebraska/South Dakota [Upper Midwest West (UMW)]. Those regions represented areas within the US characterized by high (SE and UME) and low (S and UMW) swine density
(Chase-Topping et al., 2020) [37]	5.93–32.3	Within	Generic	Product of transmission rate and duration of infection	Stochastic compartmental model	Data from an animal experiment conducted by authors (Belgium)
(Charpin et al., 2012) [38]	2.6 (CI: 1.8–3.3)	Within	Generic	Survival Function Method	Deterministic compartmental model	Data from an animal experiment conducted by authors (France)
(Rose et al., 2015) [39]	5.42 (CI: 2.94–9.04)	Within	Generic	Product of transmission rate and duration of infection	Stochastic compartmental model	Animal experiments conducted by the authors (France)
(Pileri et al., 2015) [40]	2.78 (CI: 2.13–3.43)	Within	Generic	Product of transmission rate and duration of infection	Stochastic compartmental model	Animal experiments conducted by the authors (Spain)
Encephalomyocarditis						
(Kluivers et al., 2006) [13]	1.36 (0.93–2.23)	Within	Belgium	White and Pagano Method	Stochastic compartmental model	Serology data from infected pens in a pig farm during an autumn 2001 outbreak in Belgium
(Maurice et al., 2002) [14]	1.24 (0.39–4.35)	Within	Generic	White and Pagano Method	Stochastic compartmental model	Animal experiments conducted by the authors (Belgium)
Classical Swine Fever						
(Chowdhuary et al., 2020) [41]	1.6 (CI:1.5–1.7)	Between	Bangladesh	Attack Rate Method	Exponential growth function	Primary data of identified cases of CSF during the Nov–Dec 2015 outbreak in Bangladesh
(Chowdhuary et al., 2020) [41]	1.5 (CI: 1.3–1.7)	Between	Bangladesh	Exponential Growth Method	Exponential growth function	Primary data of identified cases of CSF during the Nov–Dec 2015 outbreak in Bangladesh
(Suresh et al., 2023) [42]	1.0–2.07	Between	India	Attack Rate Method	Diverse machine Learning Algorithms such as Random Forest, Classification tree analysis, and gradient boosting	Incidence data from 2005 to 2021 from surveillance activities in India.
(Stegeman et al., 1999) [43]	6.8	Between	The Netherlands	Product of the transmission rate and duration of infection	Deterministic compartmental model	Surveillance by the authors of 21,500 herds that reported the infection between 1997 and 1998
(Stegeman et al., 1999) [44]	2.9	Within	The Netherlands	Exponential Growth Method	Stochastic compartmental model	Surveillance by the authors of 82 herds that reported the infection between 1997 and 1998
(Laevens et al., 1998) [18]	81.3 (S.E. 109.54)	Within	Generic	Martingale estimator using final outbreak size approach	Kaplan-Meier Survival Analysis	Animal experiments conducted by the authors (Belgium)
(Laevens et al., 1999) [19]	13.7 (S.E. 13.7)	Within	Generic	Martingale estimator using final outbreak size approach	Cox Proportional Hazard Analysis	Animal experiments conducted by the authors (Belgium)
(Klinkenberg et al., 2002) [45]	Within Pen: Weaner: 100 (CI: 54.4–186) Slaughter 15.5 (CI: 6.20–38.7) Between Pen: Weaner 7.77 (CI: 4.68–12.9 Slaughter 3.39 (CI: 1.54–7.45)	Within	Generic	Product of the transmission and duration of infection	Stochastic compartmental model	Animal experiments by Laevens et al. [19]
(Bouma et al., 2000) [46]	2.3 (CI:0–5.0)	Within	Generic	Product of the transmission rate and duration of infection	Stochastic compartmental model	Animal experiments conducted by the authors (The Netherlands)
(Dewulf et al., 2001) [47]	13.0	Within	Generic	Martingale estimator using final outbreak size approach	Stochastic compartmental model	Animal experiments conducted by the authors (Belgium)
(Durand et al., 2009) [48]	Within Pen: (VB) dataset 4.0 (CI: 1.9–8.4), (CB) dataset qualitative 7.6 (CI: 3.4–17.0) quantitative 12.2 (CI: 5.5–27.3) Between Pen: (VB) dataset 1.1 (CI: 0.4–2.9), (CB) dataset qualitative 1.4 (CI: 0.5–3.7), quantitative 1.8 (CI: 0.7–4.8)	Within	Generic	Product of the transmission and duration of infection	Deterministic compartmental model	Animal experiments conducted by the authors. Viraemia-based (VB), Clinical-based (CB) qualitative or quantitative. (France)
(Weesendorp et al., 2011) [49]	Low: 23.1 (CI: 11.5–45.0) High: 143 (CI: 56.3–373)	Within	Generic	Product of the transmission rate and the duration of infection	Stochastic compartmental model	Animal experiments conducted by the authors (The Netherlands)
(Weesendorp et al., 2009) [50]	Zoelen: 0 (CI: 0–0.92) Paderborn Middle: 148 (CI: 53.8–382) Paderborn High: 35.9 (CI: 14.5–77.6) Brescia: 17.5 (CI: 7.13–36.9)	Within	Generic	Product of the transmission rate and the duration of infection	Stochastic compartmental model	Animal experiments conducted by the authors (The Netherlands)
African Swine Fever						
*Genotype IX*						
(Barongo et al., 2015) [51]	1.63 (CI:1.56–1.72)	Between	Uganda	Exponential Growth Method	Epidemic doubling method	Data was from previous studies from villages in Gulu District performed by other researchers. Outbreaks included in this study occurred between April 2010 and November 2011
(Barongo et al., 2015) [51]	3.24 (CI: 3.21–3.27)	Between	Uganda	Estimated directly from model simulation	Spatial simulation model	Data was from previous studies from villages in Gulu District performed by other researchers. Outbreaks included in this study occurred between April 2010 and November 2011
(Barongo et al., 2015) [51]	1.90 (CI: 1.87–1.94)	Between	Uganda	Product of transmission rate and duration of infection	Deterministic compartmental model (model fitting using linear regression)	Data was from previous studies from villages in Gulu District performed by other researchers. Outbreaks included in this study occurred between April 2010 and November 2011
(Barongo et al., 2015) [51]	1.58	Between	Uganda	Product of transmission rate and duration of infection	Deterministic compartmental model (model fitted using curve fitting)	Data was from previous studies from villages in Gulu District performed by other researchers. Outbreaks included in this study occurred between April 2010 and November 2011
(Barongo et al., 2015) [51]	1.77 (CI: 1.74–1.81)	Between	Uganda	Product of transmission rate and duration of infection	Deterministic compartmental model (model calibrated using bootstrapping)	Data was from previous studies from villages in Gulu District performed by other researchers. Outbreaks included in this study occurred between April 2010 and November 2011
*Genotype I*						
(Eblé et al., 2019) [52]	0.3	Within	Generic	Product of the transmission rate and the duration of infection	Deterministic compartmental model	Data from lab conducted by other researcher (The Netherlands)
(de Carvalho Ferreira et al., 2013) [53]	18.0 (CI: 6.90–46.9).	Within	Generic	Product of the transmission rate and the duration of infection	Reconstructed compartmental model	Animal experiments conducted by the authors (The Netherlands)
(Korennoy et al., 2017) [54]	7.46 (CI: 5.68–9.21)	Within	Ukraine	Exponential Growth Method	The epidemic curve is modeled as an exponential growing function	General Office of Veterinary, Ministry of Agriculture, USSR, Moscow during the period from 10 February to 2 July 1977 for Odessa.
(Korennoy et al., 2017) [54]	1.65 (CI: 1.42–1.88)	Between	Ukraine	Exponential Growth Method	The epidemic curve is modeled as an exponential growing function	General Office of Veterinary, Ministry of Agriculture, USSR, Moscow during the period from 10 February to 2 July 1977 for Odessa.
*Genotype II*						
(Huang et al., 2021) [55]	0.6	Not specific	China	Next-Generation Method	Deterministic compartmental model	Ministry of Rural Agriculture of China: The cumulative number of reported infected pigs by ASF is the cumulative number of African Swine Fever cases that comes from the Official Veterinary Bulletin
(Gulenkin et al., 2011) [56]	2–3	Between	Russia	Product of the transmission rate and the duration of infection.	Deterministic compartmental model	Data for this study was acquired from WAHID (OIE) [World Animal Health Information System Office International Epizootic] database. R_0_ between herds was calculated with data from the 2008 Republic of North Ossetia.
(Mai et al. 2022) [57]	2.48 (CI: 2.39–2.58) and 4.5 (CI: 4.35–4.65), 5.4 (CI: 5.1–5.55), 1.58 (CI: 1.56–1.61), 1.41 (CI: 1.38–1.44), and 1.94 (CI: 1.92–1.97) at national and Hung Yen, Thai Binh, Thai Nguyen, Quang Ninh, Hai Duong Provinces	Between	Vietnam	Product of the transmission rate and the duration of infection.	Stochastic compartmental model	Data from early outbreak February to August 2019. Local data of ASF outbreaks in Thai Binh, Hung Yen, Thai Nguyen, Quang Ninh, and Hai Duong Provinces were reported to their respective Animal Husbandry and Veterinary sub-departments under the Department of Animal Health, Vietnam. National data for this study were collected directly from the Department of Animal Health
(Song et al., 2022) [58]	4.1865	Within	China	Next-Generation Method	Deterministic compartmental model	August 2018 ASF outbreak at an Aiyuan farm of Jiangsu Jiahua Breeding Pig Company in Siyang County, China. There are 14,929 pigs in the 13 pigpens
(Li et al., 2022) [59]	4.82–11.90	Within	China	Next-Generation Method	Compartmental model that explicitly accounts for both direct and indirect transmission between pigs. Indirect transmission driven by contaminated swills and fomites	Data collected by China Animal Health and Epidemiology Center pertaining to four outbreaks in September 2018 in Xuan Zhou
(Gulenkin et al., 2011) [56]	8–11	Within	Russia	Product of the transmission rate and the duration of infection	Deterministic compartmental model (model fitted using curve fitting)	Data acquired from WAHID (OIE) [World Animal Health Information System Office International Epizootic] database. Reported ASF outbreaks in the territory of the Russian Federation during the period 2007–2010.
(Gulenkin et al., 2011) [56]	9.8 (CI: 3.9–15.6)	Within	Russia	Product of the transmission rate and the duration of infection	Deterministic compartmental model (model calibrated using generalized linear modeling)	Data acquired from WAHID (OIE) database. Reported ASF outbreaks in the territory of the Russian Federation during the period 2007–2010.
(Guinat et al., 2018) [60]	4.4 (CrI: 2.0–13.4) 17.3 (CrI: 3.5–45.5)	Within	Russian Federation	Product of the transmission rate and the duration of infection.	Stochastic compartmental model	Incidence and testing data for ASFV in the RF are routinely collected by the Federal Research Center for Virology and Microbiology (FRCVM). Data on pig mortality were obtained for nine pig herds in which ASFV was detected through routine surveillance between 2010 and 2014
(Le et al., 2023) [61]	10.4 (CrI: 1.1–30.4)	Within	Vietnam	Production of the transmission rate and the duration of infection	Stochastic compartmental model	Surveillance outbreak data from a small, 17 pigs, family-owned farm in Thái Bình province.
(Mai et al., 2022) [62]	1.49 (CI: 1.05–2.2)	Within	Vietnam	Exponential growth Method	Deterministic compartmental model	Data collected from ten private farms categorized in different scales (seven farms from 100 to 299 and the rest from 300 to 999) were purposely included after these farms were confirmed with ASF-infected status by real-time PCR technique. Farms were selected based on the availability and quality of the data collected on morbidity. All provinces were in Northern Vietnam.
(Mai et al., 2022) [62]	1.58 (CI: 0.92–2.56)	Within	Vietnam	White and Pagano Method	Deterministic compartmental model	Data collected from ten private farms categorized in different scales (seven farms from 100 to 299 and the rest from 300 to 999) were purposely included after these farms were confirmed with ASF-infected status by real-time PCR technique. Farms were selected based on the availability and quality of the data collected on morbidity. All provinces were in Northern Vietnam.
(Mai et al., 2022) [62]	1.46 (CI: 1.38–1.57)	Within	Vietnam	Attack Rate Method	Deterministic compartmental model	Data collected from ten private farms categorized in different scales (seven farms from 100 to 299 and the rest from 300 to 999) were purposely included after these farms were confirmed with ASF-infected status by real-time PCR technique. Farms were selected based on the availability and quality of the data collected on morbidity. All provinces were in Northern Vietnam.
(Mai et al., 2022) [63]	1.55–1.78	Within	Vietnam	Product of the sum of the infection incidence and the weighted infectivity function.	A serial interval model based on daily incidence assuming a Poisson distribution.	Two commercial farrow-to-finish pig farms (HY1 and HY2) located in two different districts in Hung Yen province, Vietnam were selected immediately after ASF outbreaks were confirmed in the two farms.
(Guinat et al., 2016) [64]	Model 1: 4.0 (CI: 1.2–8.5) Model 2: 5.3 (CI: 1.7–10.3) Model 3: 7.2 (CI: 2.1–14.2) Between Pen: Model 1: 2.0 (CI: 1.2–8.5) Model 2: 2.5 (CI: 0.8–5.2) Model 3: 3.5 (CI: 1.2–7.0)	Within	Generic	Product of the transmission rate and the duration of infection	Deterministic compartmental model	Animal experiments conducted by the authors. (United Kingdom)
(Oh et al., 2023) [65]	2.91 (CI: 1.51–7.06)	Within	Generic	Exponential growth Method	Survival Analysis	Animal experiments conducted by the authors (Vietnam)
(Oh et al., 2023) [65]	4.015 (CI:1.13–9.8)	Within	Generic	White and Pagano Method	Survival Analysis	Animal experiments conducted by the authors (Vietnam)
*Genotype Not Specified*						
(Chuchard et al., 2022) [66]	18.57	Between	Generic	Next-Generation Method	Deterministic compartmental model	None
(Kouidere et al., 2021) [67]	5.71	Within	Generic	Next-Generation Method	Deterministic compartmental model	None
(Shi et al., 2023) [68]	3.88–9.92	Within	Generic	Next-Generation Method	Fractional-order Compartmental model	None
(Shi et al., 2023) [69]	13.02	Within	Generic	Next-Generation Method	Fractional-order Compartmental model	None
(Shi et al., 2020) [70]	3.77	Within	Generic	Next-Generation Method	Fractional-order Compartmental model	None

IQR: Interquartile range; CI: 95% Confidence Interval; CrI: 95% Credible Interval.

## Data Availability

No new data were created or analyzed in this study. Data sharing is not applicable to this article.
